# Effectiveness of anisodamine for the treatment of critically ill patients with septic shock: a multicentre randomized controlled trial

**DOI:** 10.1186/s13054-021-03774-4

**Published:** 2021-09-27

**Authors:** Yuetian Yu, Cheng Zhu, Yucai Hong, Lin Chen, Zhiping Huang, Jiancang Zhou, Xin Tian, Dadong Liu, Bo Ren, Cao Zhang, Caibao Hu, Xinan Wang, Rui Yin, Yuan Gao, Zhongheng Zhang

**Affiliations:** 1 Department of Critical Care Medicine, Ren Ji Hospital, School of Medicine, Shanghai Jiao Tong University, Shanghai, People’s Republic of China; 2 Department of Disease Prevention and Control, Rui Jin Hospital, School of Medicine, Shanghai Jiao Tong University, Shanghai, People’s Republic of China; 3 Department of Emergency Medicine, Rui Jin Hospital, School of Medicine, Shanghai Jiao Tong University, Shanghai, People’s Republic of China; 4 Department of Emergency Medicine, Sir Run Run Shaw Hospital, Zhejiang University School of Medicine, No 3, East Qingchun Road, Hangzhou, 310016 Zhejiang Province People’s Republic of China; 5 Department of Critical Care Medicine, Affiliated Jinhua Hospital, Zhejiang University School of Medicine, Jinhua, People’s Republic of China; 6 Department of Critical Care Medicine, Beilun District People’s Hospital, Zhejiang Province, Ningbo, People’s Republic of China; 7 Department of Critical Care Medicine, Sir Run Run Shaw Hospital, Zhejiang University School of Medicine, Hangzhou, 310016 People’s Republic of China; 8 Department of Critical Care Medicine, Lishui Municipal Central Hospital, Lishui, 323000 People’s Republic of China; 9 Department of Critical Care Medicine, Affiliated Hospital of Jiangsu University, Zhenjiang, People’s Republic of China; 10 Department of Critical Care Medicine, The First People’s Hospital of Yongkang Affiliated To Hangzhou Medical College, Jinhua, 321300 People’s Republic of China; 11 Department of Critical Care Medicine, Taizhou Hospital of Zhejiang Province Affiliated To Wenzhou Medical University, Taizhou, People’s Republic of China; 12 Department of Intensive Care Medicine, Zhejiang Hospital, Hangzhou, 310000 Zhejiang People’s Republic of China; 13 Department of Intensive Care Medicine, Binzhou Maternal and Child Health Care Hospital, Binzhou, Shandong People’s Republic of China; 14 Department of Intensive Care Medicine, Binzhou People’s Hospital Affiliated To Shandong First Medical University, Binzhou, Shandong People’s Republic of China

**Keywords:** Septic shock, Anisodamine, Randomized controlled trial, Mortality, Mechanical ventilation

## Abstract

**Background:**

Septic shock is characterized by an uncontrolled inflammatory response and microcirculatory dysfunction. There is currently no specific agent for treating septic shock. Anisodamine is an agent extracted from traditional Chinese medicine with potent anti-inflammatory effects. However, its clinical effectiveness remains largely unknown.

**Methods:**

In a multicentre, open-label trial, we randomly assigned adults with septic shock to receive either usual care or anisodamine (0.1–0.5 mg per kilogram of body weight per hour), with the anisodamine doses adjusted by clinicians in accordance with the patients’ shock status. The primary end point was death on hospital discharge. The secondary end points were ventilator-free days at 28 days, vasopressor-free days at 28 days, serum lactate and sequential organ failure assessment (SOFA) score from days 0 to 6. The differences in the primary and secondary outcomes were compared between the treatment and usual care groups with the *χ*^2^ test, Student’s t test or rank-sum test, as appropriate. The false discovery rate was controlled for multiple testing.

**Results:**

Of the 469 patients screened, 355 were assigned to receive the trial drug and were included in the analyses—181 patients received anisodamine, and 174 were in the usual care group. We found no difference between the usual care and anisodamine groups in hospital mortality (36% vs. 30%; *p* = 0.348), or ventilator-free days (median [Q1, Q3], 24.4 [5.9, 28] vs. 26.0 [8.5, 28]; *p* = 0.411). The serum lactate levels were significantly lower in the treated group than in the usual care group after day 3. Patients in the treated group were less likely to receive vasopressors than those in the usual care group (OR [95% CI] 0.84 [0.50, 0.93] for day 5 and 0.66 [0.37, 0.95] for day 6).

**Conclusions:**

There is no evidence that anisodamine can reduce hospital mortality among critically ill adults with septic shock treated in the intensive care unit.

*Trial registration* ClinicalTrials.gov (NCT02442440; Registered on 13 April 2015).

## Background

Sepsis is a leading cause of morbidity and mortality in the intensive care unit (ICU), and its severe form, septic shock can have a mortality rate as high as 40% [1]. A recent epidemiological study shows that the global annual incidence of sepsis is approximately 50 million and that sepsis-related deaths reach 11 million in 2017, representing 19.7% of all global deaths [2]. Given the high global burden of sepsis, great efforts have been made to improve its clinical outcomes. Interventions such as early goal-directed therapy, resuscitation bundles, protective ventilation, high-volume haemofiltration and immunomodulatory agents have been widely explored [3–7] and significant improvements in sepsis management and outcomes have been witnessed over the past few decades [8, 9]. However, sepsis-related mortality and morbidity are still unacceptably high as estimated from the global disease burden database [2], and exploring novel therapeutic agents is a top research priority for sepsis/septic shock [10].

The primary underlying pathophysiology of septic shock is microcirculatory dysfunction, which in turn leads to tissue hypoxia, organ dysfunction and even mortality. In this regard, sepsis is also defined as a severe endothelial dysfunction syndrome that develops in response to infections leading to reversible or irreversible injury to the microcirculation, which is responsible for multiple organ failure [11]. Therefore, one of the most important approaches for the successful treatment of septic shock is to ameliorate the uncontrolled inflammatory response and endothelial injury. Anisodamine has been shown to be effective in improving microcirculation and reperfusion injuries by reducing oxidative stress, apoptosis and inflammatory responses [12, 13], as well as by the activation of the cholinergic anti-inflammatory pathway [14]. Anisodamine has been widely used in China since 1965 for the treatment of circulatory disorders such as septic shock and disseminated intravascular coagulation (DIC). However, the quality of evidence supporting the use of anisodamine in septic shock is low [15–17]. Thus, we conducted a randomized controlled trial to test the efficacy of anisodamine in septic shock. We hypothesized that anisodamine was able to reduce hospital mortality for critically ill patients with septic shock.

## Methods

### Study design and setting

This was an open-label randomized controlled trial conducted in 12 tertiary care hospitals from May 2015 to October 2020. The study protocol has been described elsewhere [18]. The study was significantly delayed due to the slow accrual of participants and the outbreak of coronavirus disease (COVID)-19 pandemic. Investigators in each participating centre screened patients with septic shock for potential eligibility. The study was reviewed and approved by the institutional review board (IRB) of each participating hospital and ethical approvals were obtained from each hospital. Informed consent was obtained from participants or their next of kin. The study was registered on the ClinicalTrials.gov website (registration No.: NCT02442440). The study was conducted in accordance with the Helsinki Declaration for clinical trials involving human subjects.

### Participants

Subjects with septic shock were considered potentially eligible for the study. Sepsis was defined in accordance with the Sepsis-2.0 criteria [19]. Patients with documented/suspected infection plus systemic inflammatory response syndrome (SIRS) were eligible. SIRS was diagnosed in patients who met at least two of the following 4 criteria for a systemic inflammatory response: (1) white blood cell count > 12,000 or < 4000 or > 10% band forms; (2) body temperature > 38 °C (any route) or < 36 °C (core temperatures only, via indwelling catheter, esophageal, rectal routes); (3) heart rate (> 90 beats/min) or use of medications that slow heart rate or paced rhythm; and (4) tachypnea (> 20 breaths per minute) or an arterial partial pressure of carbon dioxide less than 4.3 kPa (32 mmHg). Suspected or documented infection of the following sites was considered: blood, lower respiratory tract, urinary tract, abdomen, skin and soft tissue, and central nervous system.

Septic shock was defined as sustained arterial hypotension with a systolic blood pressure (SBP) < 90 mmHg, mean arterial pressure (MAP) < 70 mmHg, or a decrease in SBP > 40 mmHg, despite adequate fluid resuscitation. The exclusion criteria were as follows: (1) age < 15 years; (2) moribund (expected to die within 24 h); (3) stay in the ICU exceeding 24 h at enrollment; and (4) contraindications to anisodamine, including acute phase of intracranial haemorrhage, elevated intracranial pressure, enlargement of prostate without urinary catheterization, glaucoma, and untreated bowel obstruction (surgically treated obstruction was not considered a contraindication).

### Interventions

The enrolled subjects were randomly assigned to receive either anisodamine or usual care. In the treated group, a bolus of 10 mg anisodamine was given intravenously as the loading dose, followed by a dosage of 0.1–0.5 mg/kg/h via pump infusion. The maintenance dose was titrated at the discretion of the treating physician according to the patients’ microcirculation status as well as side effects. For example, the infusion rate could be increased if the serum lactate level continued to increase and capillary refilling time remained prolonged. Conversely, if the use of anisodamine resulted in a significant drop in blood pressure/tachycardia, the infusion rate could be reduced. Anisodamine was discontinued after recovery from shock (vasopressor discontinuation and normalization of serum lactate), the occurrence of significant adverse events, or death. The usual care group received conventional care that did not include the use of anisodamine. Usual care for the treatment of septic shock included fluid resuscitation, use of vasopressors, early goal-directed therapy and empirical antibiotics [20].

### Outcomes

The primary outcome was hospital mortality. The enrolled subjects were followed for the length of hospital stay. Mortality at hospital discharge was defined by the vital status at discharge. The secondary outcomes included the length of stay (LOS) in the hospital and ICU, temporal trends of serum lactate and C-reactive protein (CRP) and use of vasopressors (dopamine and norepinephrine). Organ dysfunction-free days, including continuous renal replacement therapy (CRRT), mechanical ventilation (MV) and vasopressor-free days at 28 days, were reported. Patients who requested to leave the hospital, gave up treatment or was transferred to another hospitals before day 28 were followed for up to 28 days.

Several major adverse events related to anisodamine administration (bowel obstruction, urine retention, tachycardia and arrythmia) were pre-specified in the case report form and were screened daily by the investigators. Other minor adverse events including but not limited to dry mouth, flushing, mild mydriasis, and blurred near vision were reported by the clinicians in charge if any of them were suspected to be associated with anisodamine use.

### Randomization and blinding

Blocked randomization was performed where anisodamine or usual care was allocated at random in a ratio of 1:1 in blocks of sizes 2, 4, 6, 8, and 10 for 355 subjects. An advantage of small block sizes (such as block size = 2) is that treatment group sizes are very similar. However, the disadvantage is that it is possible to guess some allocations, thus reducing blinding in the trial. A solution is to use random sequences of block sizes so that the allocations cannot be guessed [21]. Central randomization was performed to ensure allocation concealment. After enrolment, investigators at each participating hospital contacted the allocation centre for a sequence number, and the participant was assigned to either the treatment or usual care group.

The caregivers at each hospital were aware of the treatment assignments. However, the investigators who assessed the outcomes and the technicians who performed the laboratory tests were blinded to the treatment assignments.

### Statistical analysis

An asymmetric two-sided group sequential design was adopted with binding futility bounds, 6 analyses, a sample size of 355, 80% power and 5% (2-sided) type I error. The mortality rate in the usual care group was assumed to be 50%, with the new intervention reducing the mortality rate by 15%. The futility bounds were derived using a Hwang-Shih-DeCani spending function with gamma = − 2 [22]. The assumption in the study design was based on our previous work that the mortality of septic shock can be as high as 50% [1], and previous studies also showed that anisodamine could reduce mortality by more than 20% [23–25].

Descriptive analytics were performed with conventional approaches: skewed numeric variables were expressed as the median and the first interquartile (Q1) and third interquartile (Q3), and normally distributed data were expressed as the mean and standard deviation. Numerical variables were compared between the treated and usual care groups with the Student t test or rank-sum test, as appropriate. Categorical data were expressed as percentages and compared between groups using the *χ*^2^ test [26].

A log-rank test was performed to investigate whether there was a survival difference between the treated and usual care groups. The results were visualized with survival curves. Patients who were alive at hospital discharge were censored on the discharge day.

The differences in serum lactate and Sequential Organ Failure Assessment (SOFA) score between the treated and usual care groups through days 0–6 were compared using the Wilcoxon test, and *p* values were adjusted by the Bonferroni method. The requirement for any type of vasopressor, norepinephrine or dopamine was compared between the treated and usual care groups, and statistical inference was performed by univariate logistic regression models. Multiple testing for secondary outcomes was adjusted for the false discovery rate (FDR) by using the Benjamini–Hochberg method [27]. All statistical analyses were performed using R (version 4.0.1). A two-tailed *p* < 0.05 was considered statistically significant.

## Results

### Participants

**Fig. 1:**
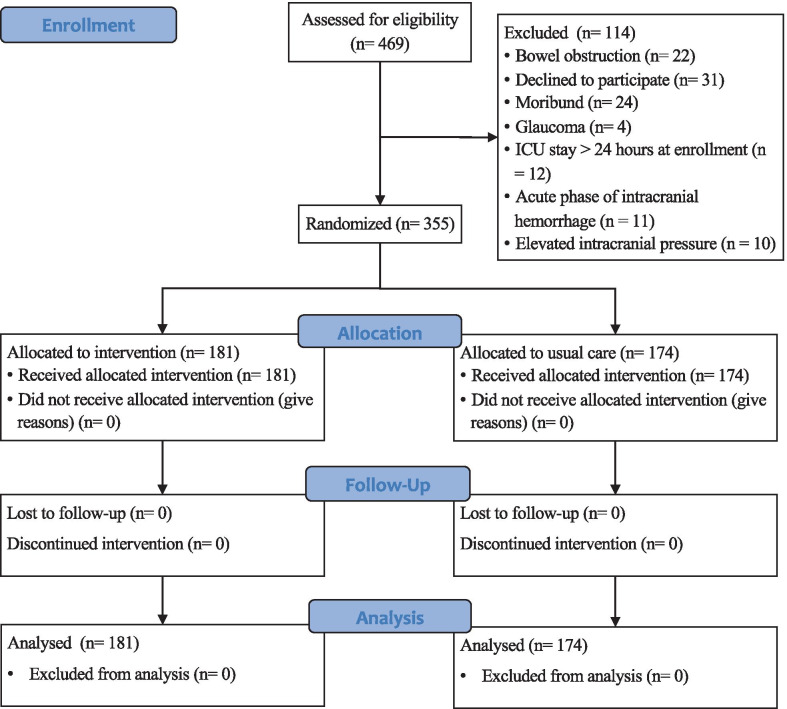
Recruitment and randomization of the patients. Patients could meet more than one exclusion criteria. *ICU* intensive care unit

A total of 469 subjects were screened from the participating hospitals, and 114 were excluded based on the exclusion criteria. Finally, 355 subjects were randomized and followed up for the duration of their hospital stay. There were 181 subjects in the treated group and 174 in the usual care group (Fig. 1). The baseline characteristics of the included patients were similar between the two groups (Table 1). There were more male patients than female patients (61% vs. 39%). The patients were mostly likely to be admitted from the ward (34%), followed by from the emergency room, postoperation and others. The most common comorbidities were diabetes and hypertension. The top two infection sites were the lower respiratory tract (33%) and the abdomen (32%). There were 177 patients (51%) who required mechanical ventilation, and 26 patients who required CRRT. The median duration of anisodamine use in the treated group was 2.8 days (IQR: 1.9–3.8 days).

**Table 1:** Baseline characteristics in the control and treated groups

Variables	Total (*n* = 355)	Control (*n* = 174)	Treated (*n* = 181)
Age (years), Median (Q1, Q3)	68 (57, 79)	67 (56, 78)	69 (58.75, 79)
Gender, male *n* (%)	217 (61)	104 (60)	113 (62)
Source of admission, *n* (%)			
Emergency room	108 (30)	52 (30)	56 (31)
Other	10 (3)	2 (2)	8 (5)
Postoperative	114 (32)	57 (33)	57 (31)
Transfer from other hospital	2 (1)	2 (1)	0 (0)
Ward	121 (34)	61 (35)	60 (33)
Type, *n* (%)			
Emergency operation	109 (31)	57 (33)	52 (29)
Medical	194 (55)	91 (52)	103 (57)
Optional operation	52 (15)	26 (15)	26 (14)
Comorbidity			
Diabetes, *n* (%)	74 (21)	40 (23)	34 (19)
Hypertension, *n* (%)	92 (26)	46 (26)	46 (26)
Myocardial infarction, *n* (%)	14 (4)	8 (5)	6 (3)
Heart failure, *n* (%)	22 (6)	11 (6)	11 (6)
Cerebrovascular, *n* (%)	29 (8)	17 (10)	12 (7)
Dementia, *n* (%)	11 (3)	6 (3)	5 (3)
COPD, *n* (%)	23 (6)	10 (6)	13 (7)
Connective tissue, *n* (%)	18 (5)	9 (5)	9 (5)
Paralysis, *n* (%)	7 (2)	4 (2)	3 (2)
Renal failure, *n* (%)	13 (4)	7 (4)	6 (3)
Malignancy, *n* (%)	59 (17)	33 (19)	26 (14)
Hematological malignancy, *n* (%)	8 (2)	4 (2)	4 (2)
Cirrhosis, *n* (%)	11 (3)	3 (2)	8 (4)
Metastatic tumor, *n* (%)	15 (4)	8 (5)	7 (4)
Immunosuppression, *n* (%)	28 (8)	17 (10)	11 (6)
Infection sites, *n* (%)			
Abdominal	114 (32)	61 (35)	53 (29)
Bile duct	24 (7)	6 (3)	18 (10)
Bloodstream	21 (6)	9 (5)	12 (7)
CNS	2 (1)	2 (1)	0 (0)
Gastrointestine	9 (3)	5 (3)	4 (2)
Hemo	4 (1)	3 (2)	1 (1)
Liver abscess	3 (1)	1 (1)	2 (1)
Mediastinum	1 (0)	0 (0)	1 (1)
Lower respiratory tract	116 (33)	63 (36)	53 (29)
Skin	10 (3)	6 (3)	4 (2)
Thoracic	3 (1)	1 (1)	2 (1)
Unknown	14 (4)	5 (3)	9 (5)
Urinary tract	34 (10)	12 (7)	22 (12)
SOFA, median (Q1, Q3)	8 (5, 11)	8 (6, 11)	8 (5, 10)
GCS, median (Q1, Q3)	14 (11, 15)	14 (11, 15)	15 (11, 15)
MV, *n* (%)	177 (51)	94 (54)	83 (47)
CRRT, *n* (%)	26 (7)	14 (8)	12 (7)

*MV* mechanical ventilation, *CRRT* continuous renal replacement therapy, *GCS* Glasgow coma scale, *SOFA* sequential organ failure assessment, *CNS* central nervous system, *COPD* chronic obstructive pulmonary disease, *Q1* first quartile, *Q3* third quartile

### Clinical outcomes of the treated and usual care groups

**Fig. 2:**
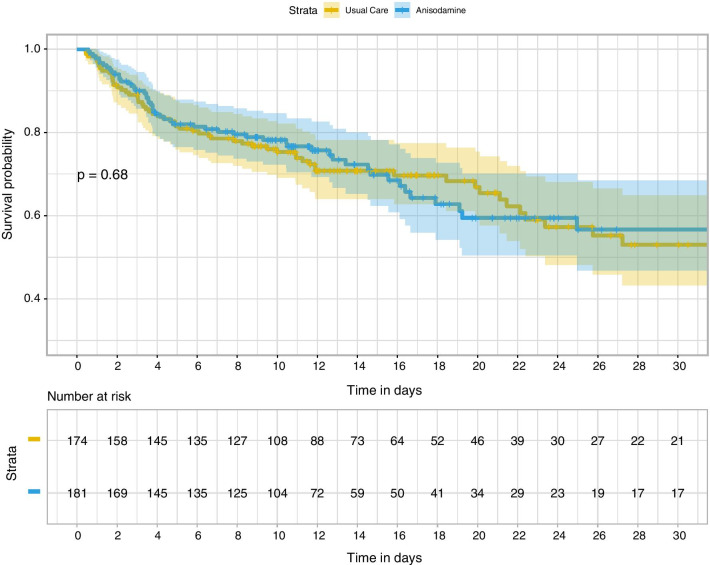
Kaplan–Meier estimates of the probability of survival to day 30. *p* value for the log-rank test was 0.68

**Fig. 3:**
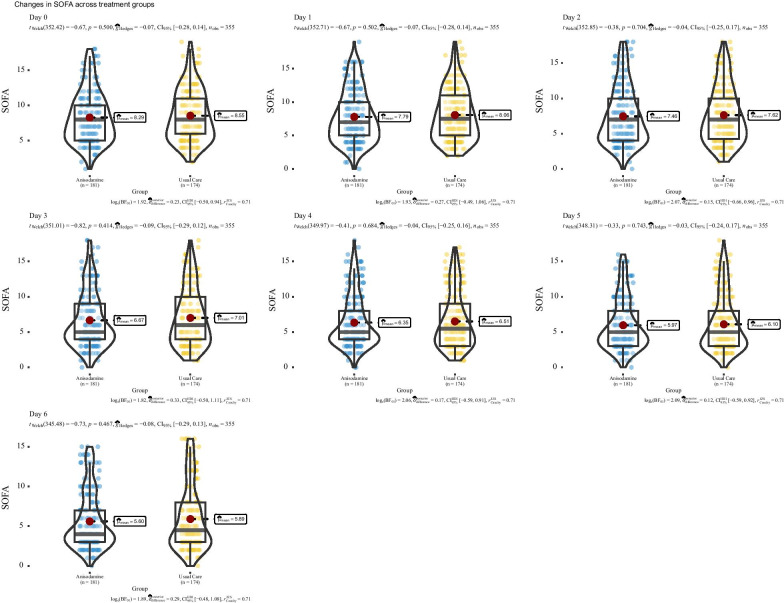
Comparisons of SOFA scores between the treated and usual care groups from days 0 to 6. The *p* values were adjusted for the false discovery rate (FDR). *SOFA* sequential organ failure assessment

The treated group showed slightly lower hospital mortality than the usual care group, but the difference was not statistically significant (36% vs. 30%; *p* = 0.348). There was no difference in ICU mortality between the two groups (22% vs. 18%; *p* = 0.397). The log-rank test comparing Kaplan–Meier survival curves did not show evidence of a survival difference between the two groups (*p* = 0.68, Fig. 2). These were no differences in the other secondary outcomes, including length of stay in the hospital (median [Q1, Q3]: 12[7.6 vs. 20.8] vs. 10.8 [5.8, 16.7] days; *p* = 0.348) and ICU (5.8 [3.3, 11.2] vs. 5.6 [3.4, 9.8] days; *p* = 0.617). The other secondary outcomes, including MV duration (5.6 [3.6, 9.9] vs. 4.8 [2.8, 9.8] days; *p* = 0.632) and CRRT days (3.5 [2.8, 8.1] vs. 7.2 [4.4, 8.9] days; *p* = 0.435) were similar between the two groups. Furthermore, we explored organ failure-free days at 28 days after enrolment. The MV, CRRT and vasopressor-free days within 28 days were not significantly different between the two groups (Table 2). No remarkable/major adverse events related to anisodamine use were reported during the study period. The SOFA scores from day 0 to day 6 were also compared between the two groups by adjusting for the FDR. The results showed that there was no significant difference between the two groups (Fig. 3).

**Table 2:** Comparison of primary and secondary clinical outcomes between the treated and control groups

Variables	Total (*n* = 355)	Control (*n* = 174)	Treated (*n* = 181)	*p* value	Adjusted *p* value*
Hospital mortality, *n* (%)	117 (33)	62 (36)	55 (30)	0.348	0.621
ICU mortality, *n* (%)	72 (20)	39 (22)	33 (18)	0.397	0.621
ICU LOS, median (Q1, Q3)	5.74 (3.37, 10.46)	5.79 (3.34, 11.17)	5.6 (3.39, 9.8)	0.617	0.632
Hospital LOS, median (Q1, Q3)	11.62 (6.61, 18.51)	12.01 (7.63, 20.76)	10.83 (5.81, 16.65)	0.129	0.621
Duration of vasopressor use, median (Q1, Q3)	2.69 (1.61, 4.02)	2.74 (1.71, 4.05)	2.39 (1.31, 3.79)	0.216	0.621
CRRT days, median (Q1, Q3)	4.35 (2.97, 8.85)	3.45 (2.84, 8.1)	7.17 (4.35, 8.85)	0.435	0.621
MV duration, median (Q1, Q3)	5.05 (2.99, 9.9)	5.61 (3.62, 9.92)	4.84 (2.88, 9.75)	0.632	0.632
Vasopressor free days in 28 days, median (Q1, Q3)	25.23 (10.15, 28)	25.22 (8.6, 27.94)	25.23 (12.6, 28)	0.585	0.621
MV free in 28 days, median (Q1, Q3)	25.26 (7.18, 28)	24.37 (5.86, 28)	25.99 (8.47, 28)	0.411	0.621
CRRT free in 28 days, median (Q1,Q3)	28 (11.48, 28)	28 (10.22, 28)	28 (13.42, 28)	0.366	0.632

*MV* mechanical ventilation, *LOS* length of stay, *ICU* intensive care unit, *CRRT* continuous renal replacement therapy, *Q1* first quartile, *Q3* third quartile

*p values were adjusted for false discovery rate by the Benjamini–Hochberg method

### Post hoc analysis

**Fig. 4:**
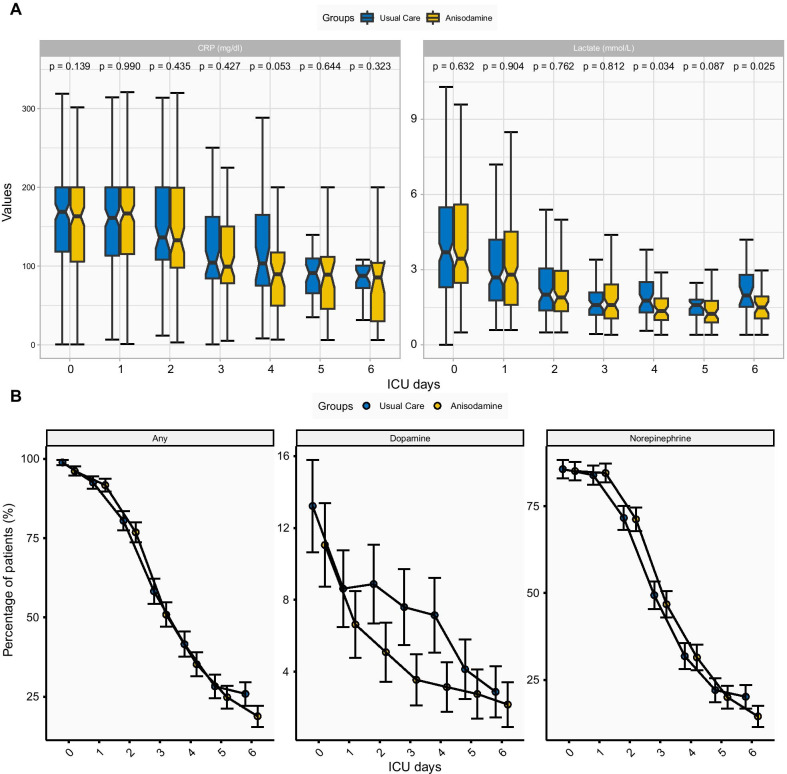
Comparisons of lactate levels, CRP levels and vasopressor requirements between the treated and usual care groups. **a** Differences in lactate and CRP levels between the two groups over time; the error bar indicates the 95% confidence interval. **b** Percentage of patients requiring vasopressors. The error bar indicates the 95% confidence interval for the percentage. The *p* values were adjusted for a false discovery rate of 0.05. *CRP* C-reactive protein

Next, we tested the hypothesis that anisodamine might improve microcirculation in patients with septic shock. Elevated serum lactate is the result of anaerobic metabolism and can be an indicator of compromised microcirculation [28]. First, the temporal trends of serum lactate were compared between the anisodamine and usual care groups (Fig. 4a). There was no significant difference between the treated and usual care groups within 3 days after enrolment. Interestingly, the treated group showed lower serum lactate levels from days 4 to 6 than the usual care group, indicating that the effect of anisodamine on microcirculation function had a late onset. We further examined the effect of anisodamine on CRP and found that starting from day 1, the treated group had lower CRP than the usual care group, but the difference was not statistically significant (Fig. 4a).

The vasopressor requirements from days 0 to 6 were compared between the two groups (Fig. 4b). The requirement for any type of vasopressor was lower on days 5 and 6. The requirement for dopamine was lower in the treated group starting from day 1, and the requirement for norepinephrine was lower in the treated group starting from day 3 (Fig. 4b). To test the statistical significance, logistic regression models were built with the use of vasopressors as the response variable and group as the independent variable. The results showed that the treated group used fewer vasopressors of any type, dopamine and norepinephrine; and the effects were more prominent on later days (Table 3).

**Table 3:** Use of vasopressors over time between the treated and control groups

	Any vasopressor		Dopamine		Epinephrine	
	OR [95% CI]	*p*	OR [95% CI]	*p*	OR [95% CI]	*p*
Day 0	0.29 [0.04, 1.22]	0.125	0.82 [0.43, 1.55]	0.532	0.96 [0.53, 1.73]	0.884
Day 1	0.89 [0.41, 1.94]	0.776	0.75 [0.34, 0.94]	0.048	1.05 [0.59, 1.86]	0.872
Day 2	0.80 [0.48, 1.35]	0.410	0.55 [0.23, 0.91]	0.039	0.98 [0.61, 1.56]	0.933
Day 3	0.74 [0.48, 1.15]	0.183	0.45 [0.15, 0.88]	0.021	0.90 [0.58, 1.39]	0.635
Day 4	0.76 [0.48, 1.21]	0.249	0.42 [0.13, 0.82]	0.012	0.98 [0.61, 1.58]	0.944
Day 5	0.84 [0.50, 0.93]	0.041	0.66 [0.17, 2.35]	0.523	0.88 [0.50, 1.55]	0.666
Day 6	0.66 [0.37, 0.95]	0.043	0.75 [0.15, 3.46]	0.710	0.67 [0.35, 0.86]	0.022

Logistic regression models were fitted with the use of any, dopamine or epinephrine as the response variable and the treatment group as the independent variable. The models were stratified by the ICU days from 0 to 6. OR < 1 indicates lower requirement of vasopressors for the treated groups against the control group

## Discussion

Our study examined the effectiveness of anisodamine in critically ill patients with septic shock. Unfortunately, the study failed to identify any beneficial effects of anisodamine in reducing the hospital mortality rate, as well as improving the other predefined clinical outcomes, including LOS in the hospital and ICU. However, we found that anisodamine was able to improve microcirculation in patients with septic shock, as supported by lower serum lactate levels and less vasopressor requirements in the treated group. Nevertheless, we noticed that the mortality rate in the treated group was lower than that in the usual care group, suggesting that the nonsignificant finding might be attributable to the limited sample size of the current study. In our study, we hypothesized that the mortality could be reduced by 15% from 50%, which represents a large beneficial effect in critical care setting. In this regard, the study is underpowered to detect the smaller beneficial effects of anisodamine. Therefore, our study hypothesized that anisodamine has a potential beneficial effect in patients with septic shock. The results need to be confirmed in future trials with greater statistical power.

The effectiveness of anisodamine has been explored in other clinical conditions with inflammatory responses. In patients with myocardial infarction, the use of anisodamine was found to be associated with improved microcirculatory perfusion and fewer inflammatory responses [29–31]. Chai and colleagues explored the effects of anisodamine in the prevention of sepsis in burn patients and found that anisodamine use was associated with 50% reduction in the incidence of sepsis in severely burned patients. They further demonstrated that the beneficial effects were mediated via the restoration of intestinal circulation [23]. In patients with acute lung injury, high-dose anisodamine was able to improve the lung function [32]. However, the potential beneficial effects of anisodamine on sepsis have mainly been explored in animal experiments. Thus, clinical trials are urgently needed to translate these findings into clinical benefits. Our study fills the gap between laboratory results and clinical effectiveness. However, since septic shock comprises a heterogeneous population, the mean effect size of the population may not be as large as expected, and the estimated sample size is actually under powered.

The anti-shock effects of anisodamine are proposed to be mediated by activating the cholinergic anti-inflammatory pathway [14]. Anisodamine blocks muscarinic receptors, which results in rerouting of acetylcholine to the α7 nicotinic acetylcholine receptor (α7nAChR) bringing about increased acetylcholine-mediated activation of α7nAChR and the cholinergic anti-inflammatory pathway [33]. This effect is supported by our observations that the treated group had lower serum lactate levels and required fewer vasopressors than the usual care group.

There are several limitations in the study that must be acknowledged. First, the study was designed as an open-label trial in which the investigators knew the group membership after treatment assignments. The risk of co-intervention imbalance cannot be fully excluded in the study. However, the outcome assessors and laboratory technicians did not know the treatment assignment. Since the clinical outcomes in our study were objective, the results were less likely to be affected by the open-label design. Second, the study is underpowered to detect a smaller-than-expected clinical effect. The best practice is to include more patients when an underpowered analysis is confirmed and a post hoc power calculation should be performed. However, the limited funding resources and slow patient recruitment did not allow us to continue the study. Future trials with larger sample sizes and more homogeneous populations can help to confirm our preliminary results. Third, the study included patients with the most severe form of sepsis, septic shock, as the study population. This target population has the highest mortality rate, which was expected to maximize the statistical power. However, it is possible that the effect size of anisodamine may be greater in patients with less severe sepsis. Finally, the study did not report the time-varying dosage of anisodamine because limited human resources prohibited the establishment of a high-granularity database.

## Conclusion

In conclusion, in critically ill adults with septic shock who were being treated in the intensive care unit, hospital mortality did not differ between patients who received anisodamine and those who received usual care without anisodamine.

## Data Availability

The information was not publicly available according to the local law.

## Abbreviations

ICU: Intensive care unit
DIC: Disseminated intravascular coagulation
IRB: Institutional review board
SIRS: Systemic inflammatory response syndrome
MAP: Mean arterial pressure
SOFA: Sequential organ failure assessment
CRRT: Continuous renal replacement therapy
LOS: Length of stay
